# Mapping the genomic landscape of antibiotic susceptiblity in *Escherichia coli*

**DOI:** 10.1038/s44259-026-00227-2

**Published:** 2026-06-04

**Authors:** Sarah Bastkowski, A. Keith Turner, Muhammad Yasir, Caroline Jarvis, Ian Charles, Mark Webber

**Affiliations:** 1 Quadram Institute Bioscience, Norwich Research Park, Norwich, UK; 2 Centre for Microbial Interactions, Norwich Research Park, Norwich, UK; 3 Norwich Medical School, University of East Anglia, Norwich, UK

**Keywords:** Computational biology and bioinformatics, Microbiology

## Abstract

Antibiotic treatment failure is frequently driven by bacterial insusceptibility, yet understanding of the full repertoire of genes contributing to this in Gram-negative bacteria remains incomplete. We used TraDIS-Xpress, a high-density transposon mutagenesis approach, to systematically define the genetic and physiological determinants of *Escherichia coli* K-12 susceptibility to 13 antibiotics spanning all major drug classes and multiple concentrations. Beyond known resistance determinants, our data reveal that the *E. coli* core genome plays a central role in modulating susceptibility. Disruption of metabolic, energy-generating, growth-associated, and amino acid biosynthetic pathways commonly enhanced survival under antibiotic stress, indicating that reduced metabolic activity and growth modulation broadly promote survival during exposure to bactericidal drugs. In contrast, pathways involved in DNA replication, peptidoglycan and lipopolysaccharide synthesis, aminoacyl-tRNA biosynthesis, riboflavin metabolism, and terpenoid backbone biosynthesis consistently aided survival. Experimental validation using defined mutants confirmed that disruption of selected metabolic and respiratory pathways confers survival benefits across multiple antibiotic classes. Together, this work provides a genome-scale framework for understanding antibiotic susceptibility in *E. coli*, identifies conserved physiological pathways underpinning survival, and offers new opportunities for strategies aimed at reversing insusceptibility and improving antibiotic efficacy.

## Introduction

The escalating crisis of treatment failure of bacterial infections due to antimicrobial resistance poses a great challenge to modern medicine and society globally. Bacterial insusceptibility to antibiotics can be due to ‘resistance’ or ‘tolerance’ and both phenotypes contribute to treatment failure^1^. Antibiotic resistance is the ability of a bacterial population to replicate in the presence of an antibiotic at a concentration above a defined clinical breakpoint which will usually be above the minimum inhibitory concentration (MIC) required to inhibit wild-type strains of that species^2^. Resistance is often a result of gain of a specific resistance gene, or mutation within the chromosome of the target bacterium and as a result is inherited by progeny of the resistant organism^3^.

Tolerance can be defined as the ability of an antibiotic susceptible microorganism to survive transient periods of exposure to high concentrations of bactericidal antibiotics^2^. Tolerance is often linked with slow growth of the target organism, and this can be caused by either environmental conditions, or mutation within cellular pathways. As a result, a tolerant phenotype may or may not be heritable. Antibiotic tolerance is now recognised as being associated with treatment failure and relapse in some settings (e.g rifampicin tolerance in Staphylococci causing joint infection^4^) although the relative contribution of tolerance versus persistence in infection remains uncertain. Tolerance also remains relatively poorly characterised from a mechanistic point of view^5^. Understanding and combating tolerance is vital for enhancing the efficacy of antibiotic therapies and addressing the broader issue of antibiotic resistance^6^.

Recent genome-wide studies examining responses to individual antibiotic classes have shown that the inhibitory action of many antibiotics is influenced by a broad range of non-target genes that contribute to downstream effects following target inhibition^7–9^. TnSeq has been used to identify genes involved in susceptibility to multiple antibiotics in Enterococcus^10^ and Streptococcus^11^ and similar studies in *Escherichia coli* have also identified genetic determinants of survival under antibiotic exposure.The *E. coli* studies have identified mechanisms underlying persister-mediated tolerance, although these have largely focused on specific antibiotics or conditions^12^. Together, these findings highlight the importance of the ‘secondary resistome’ in shaping baseline susceptibility, for example through modulation of envelope permeability^12–14^, and in influencing evolutionary trajectories towards resistance.

A recent genome-wide approach mapped genes and pathways that influence susceptibility to different antibiotics in *Streptococcus pneumoniae*. The study gave insight into the genetic pathways and cellular processes underlying both antibiotic tolerance and resistance by creating a genetic interaction network^11,15^. Whilst this study provided important new information, mechanisms underpinning antibiotic tolerance may vary across bacterial strains and species. Including Gram-negative species in similar studies is therefore crucial to advance knowledge of tolerance mechanisms, particularly given that most of the most critical pathogens where AMR is a challenge are Gram-negative.

Despite growing recognition of the interplay between multiple genes and pathways being important in determining the overall susceptibility of a bacterium to an antibiotic, the full repertoire of genes in Gram-negative bacteria which can influence this remains incompletely understood. High-resolution mutagenesis approaches have begun to illuminate antibiotic responses, and offer a route to systematically compare susceptibility determinants to multiple antibiotic classes in parallel experiments. The TraDIS-Xpress approach provides an opportunity to identify both loss-of-function and altered-expression phenotypes via the use of outward facing promoters in the transposons^15,16^, enabling comprehensive mapping of the pathways that shape antibiotic survival and influence trajectories toward antimicrobial resistance.

In this work we used TraDIS-Xpress to identify genes and pathways of *E. coli* K-12 involved in response to exposure to 13 antibiotics of all major classes across a range of concentrations representing sub- and supra-MIC levels. We used gene enrichment analysis to identify pathways that play crucial roles in responding to specific antibiotics whilst uncovering commonalities and differences reflecting varying antibiotic modes of action. This work identifies the diverse molecular mechanisms impacting antibiotic susceptibility in *E. coli* and highlights the importance of core cellular pathways in facilitating survival to antibiotic exposure.

## Results

From the TraDIS experiments an average of 3.8 million reads corresponding to transposon inserts were mapped against the BW25113 reference genome with at least ~ 0.4 million unique insertion sites recovered per sample (corresponding to recovery of an insertion mutant at an average of 9–10 bps across the genome).

The numbers of mutants recovered did vary with generally fewer reads recovered after exposure to higher concentrations of antibiotics, relating to fewer mutants surviving in these conditions. A detailed summary of mapped reads and unique insertion sites for each sample is given in Supplementary table 2. We used the insertion index (insertion sites per gene divided by gene length), calculated per gene for each sample to assess consistency between replicates which was universally high as seen in PCA plots (Supplementary Fig 1).

We performed comparisons of mutant survival between controls and conditions and then employed gene enrichment analysis to identify gene sets that were grouped based on their participation in the same biological function or pathway. Normalised enrichment scores (NES) and enrichment scores (ES) were used to give a measure of the involvement of a pathway for survival in a particular condition.

**Fig. 1:**
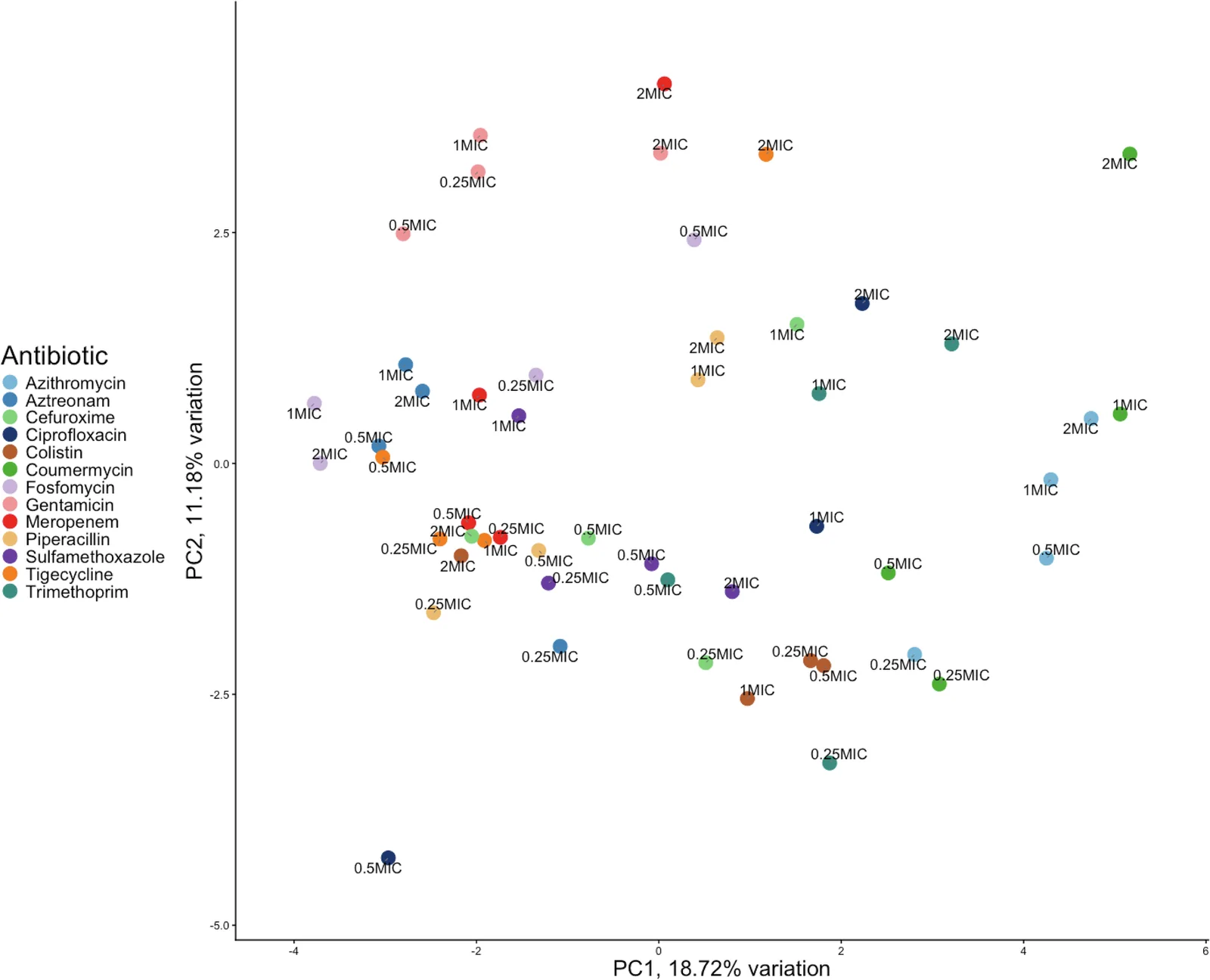
Comparisons of pathway enrichments in a large transposon mutant collection after treatment with different antibiotics. PCA plot grouping responses to each condition based on pathway enrichment (normalised enrichment score).

Figure 1 shows an overview of the similarity of responses to each condition as a PCA plot, based on pathway enrichment. This analysis identified different drugs where responses were similar even though they exert different mechanisms of action, for example aztreonam and tigecycline cluster together. Responses to other antibiotics were very different and some were distinct to any others–e.g. gentamicin clustered away from most of the other conditions, and exposure to the highest concentration of meropenem was also very distinct. For some drugs exposure resulted in responses that clustered together regardless of concentration (e.g. gentamicin) whereas others demonstrated a marked diversity in responses at different exposure concentrations. For example, coumermycin showed a binary response between high and low exposures, and azithromycin demonstrated a gradient of responses according to concentration.

**Fig. 2:**
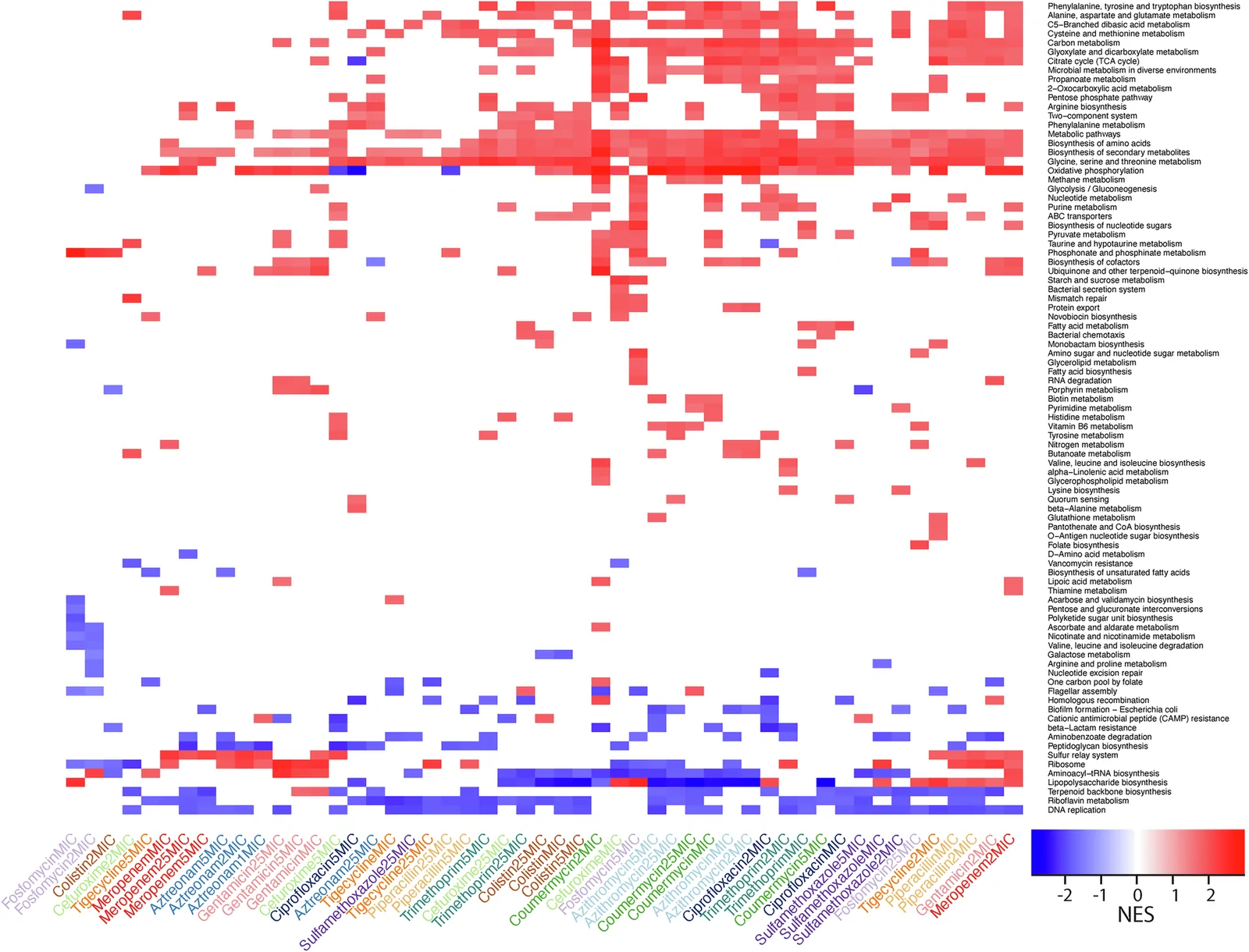
Pathways sensitive to antibiotic exposure. Heatmap of enriched pathways after exposure to 13 different antibiotics, each at 4 different concentrations (0.25, 0.5, 1 and 2X the MIC). Antibiotics are named along the horizontal axis, with each antibiotic a different colour to assist with identification. The vertical -axis shows the different pathways that were enriched or depleted significantly in at least one antibiotic condition.

To explore the mechanisms underpinning the different responses to each antibiotic we identified pathways in which mutations conferred a selective advantage or disadvantage based on enrichment scores. We clustered all the data in a heatmap where pathways were coloured by positive or negative NES. Figure 2 shows the relationships between all the antibiotics tested at multiple concentrations and enrichment or depletion of pathways based on NES. This analysis complemented the PCA analysis but more easily visualised the number of pathways that impacted fitness on different exposure conditions varied. For example, cefuroxime (at 2X MIC), tigecycline (at 1X and 2X MIC), fosfomycin and aztreonam (at 1X and 2X MIC) showed a small complement of pathways under selective pressure. This correlates with the small number of viable cells in these exposure conditions, limiting the landscape of options where mutations can help benefit against these relatively high levels of stress.

Our data represents the aggregated fitness impacts of all individual mutants within the mixed transposon mutant population, where each mutant is in competition with all others. As we would expect we identified many cellular pathways and large numbers of individual genes as being involved in responding to antibiotic stress. In general, there were more pathways within which disruption conferred a selective advantage upon antibiotic exposure than where disruption was detrimental. Therefore, loss of pathway function was more commonly associated with reduced susceptibility (Fig. 2).

As expected, the data revealed multiple examples where antibiotics selected for mutations in genes coding for their respective targets, e.g. gentamicin selected for mutants in genes coding components of the ribosomes and colistin selected for mutants in genes coding for lipopolysaccharide biosynthesis. There was however no universal response seen for all the antibiotics tested with no correlation between all the antibiotics and any of the metabolic pathways tested (Fig. 2).

Generally, disruption of pathways connected to metabolism, growth, energy, and amino acids reduced susceptibility to the 13 antibiotics (Fig. 2), whilst disruption to DNA replication, peptidoglycan synthesis, lipopolysaccharide synthesis, aminoacyl-tRNA biosynthesis, riboflavin metabolism and terpenoid backbone biosynthesis increased susceptibility.

Some pathways only impacted susceptibility for a single antibiotic, for example ascorbate/alderate metabolism mutants were depleted only by fosfomycin. Fosfomycin at 1 and 2X MIC also tended to impact a relatively small number of pathways compared to some other antibiotics that exerted a selective impact on many genes and pathways.

**Fig. 3:**
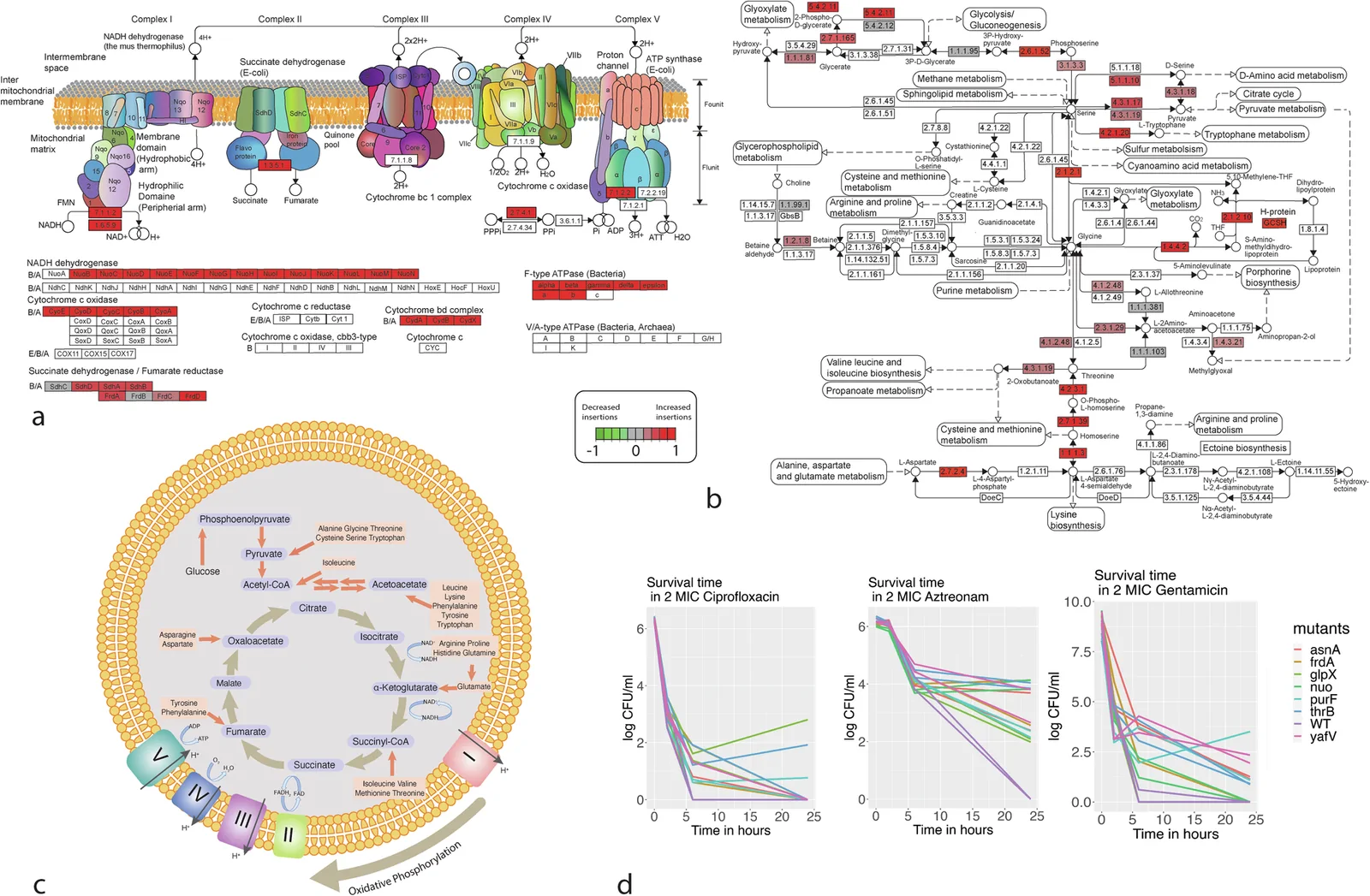
Pathways where disruption improved survival against multiple antibiotics. **a** Overview of components of oxidative phosphorylation where disruption impacted susceptibility. Genes in this pathway are annotated by a change in insertions seen after exposure to gentamicin. **b** Genes involved in glycine, serine and threonine metabolism, where disruption (shown in red) conferred a selective advantage (example data after trimethoprim exposure). **c** Overview of the TCA cycle and oxidative phosphorylation and metabolic links to amino acid biosynthesis. **d** Defined mutants of BW25113 lacking selected genes representing key functions in the previously described pathways showed increased survival after antibiotic treatment. The figure shows surviving numbers of each mutant over 24 h after exposure to 2X MIC of either aztreonam, ciprofloxacin or gentamicin. Data show average from three technical replicates for each mutant and the two independent mutants for each gene are represented by the same colour. Pathway illustration was adapted by the authors from KEGG pathway information.

Most individual exposure conditions resulted in a range of pathways being under selective pressure, Fig. 3 shows an example for coumermycin with a cellular overview with pathways and constituent genes identified as important after exposure to 1X MIC highlighted. After coumermycin exposure, mutants in lipid A-core biosynthesis, peptidoglycan maturation, various transfer RNAs and several transport pathways were depleted indicating a requirement to maintain these functions. In contrast, disruption to other pathways was beneficial. These included pathways involved in central metabolism and growth, with functions in metabolic regulation as well as fermentation and respiration. Disruption was also beneficial in pathways for the synthesis and degradation of cofactors, carriers, vitamins, amino acids, nucleosides, nucleotides, carbohydrates, alcohols, aromatic compounds, aldehydes, fatty acids, and lipids. Similar patterns were seen for many antibiotics illustrating the wide variety of pathways under selective pressure from the panel of antibiotics tested.

Some pathways, such as nucleotide salvage or amino acid biosynthesis, were biphasic responses—being beneficial when disrupted at low antibiotic concentrations, but protective at higher doses. This shows complex, condition-specific roles for certain pathways, potentially reflecting shifting cellular priorities under different levels of antibiotic stress.

### Disruption of pathways for amino acid metabolism, carbon metabolism and oxidative phosphorylation conferred a selective advantage to multiple antibiotics

Although there was no single pathway implicated in survival to all antibiotics there were a series of pathways which did contribute to survival against multiple antibiotics. The pathway with the highest positive enrichment score in most antibiotics (Fig. 2) was glycine, serine and threonine metabolism (Fig. 3b). Other amino acid metabolic pathways including phenylalanine, arginine, tyrosine and tryptophan biosynthesis and alanine, aspartate and glutamate metabolism also showed positive enrichment scores for various antibiotics indicating a general benefit upon antibiotic exposure resulting from loss of amino acid synthesis or metabolism. Other pathways where disruption conferred a selective advantage were involved in energy generation and included oxidative phosphorylation and carbon metabolism including the citrate (TCA) cycle and other energy and metabolism related pathways (Fig. 3a, b).

Figure 3b shows glycine, serine and threonine metabolism genes annotated with changes of transposon mutants after exposure to trimethoprim. Increased mutations were observed across most of the pathway, indicating that its disruption is beneficial for survival under trimethoprim exposure. This observation was consistent across many of the tested antibiotics and concentrations (Fig. 2). We observed similar, though less consistent signals for alanine, aspartate and glutamate metabolism and phenylalanine, tyrosine, and tryptophan biosynthesis. These pathways all feed the TCA cycle (Fig. 3c) and therefore impact oxidative phosphorylation, where disruption was also predicted to benefit fitness after exposure to many antibiotics. Figure 3a shows different components of the oxidative phosphorylation pathway where independent mutants were identified after exposure to gentamicin (1X MIC). Similarly strong signals for this pathway were again observed for most antibiotics and concentrations tested. The *nuo* operon codes for the NADH dehydrogenase complex, which is the first component in the electron transport chain for the oxidation of NADH to generate the proton gradient across the cytoplasmic membrane. To assemble a functional NADH:ubiquinone oxidoreductase all *nuo* genes are required. Therefore, the disruption of one gene will lead to disruption of the whole complex^17^. Consistent with a benefit arising from degradation of NADH dehydrogenase we observed significant enrichment of insertions in nearly every *nuo* gene (Fig. 3a, Supplementary Fig 2).

### Experimental validation of pathways predicted to impact susceptibility

As our data predicted the disruption of amino acid biosynthesis, the TCA cycle or oxidative phosphorylation and other pathways would all provide some benefit in the presence of antibiotics, we measured survival of mutants from the Keio collection chosen to have loss of function of one of seven pathways identified in our data (Table 1). The selected mutants were chosen to test disruption of glycine, serine and threonine metabolism (*thrB*), purine metabolism (*purF*), glycolysis (*glpX*) and alanine/aspartate/glutamate metabolism (*yafV*), both feeding the TCA cycle, oxidative phosphorylation (*nuoA* and *frdA*) or aminoacyl t-RNA biosynthesis (*asnA*).

**Table 1:** Pathways implicated in survival to multiple antibiotics and defined deletion mutants studied

Gene	Description
*thrB*	Involved in glycine, serine and threonine metabolism
*purF*	Involved in TCA cycle and purine metabolism
*glpX*	Involved in glycolysis feeding into the TCA cycle
*yafV*	Omega amidase, involved in alanine, aspartate, glutamate metabolism feeding into TCA cycle
*nuoA*	Involved in oxidative phosphorylation (disruption NADH:ubiquinone oxidoreductase (complex I))
*frdA*	Involved in oxidative Phosphorylation (disruption of fumarate reductase flavoprotein subunit)
*asnA*	Involved in aminoacyl t-RNA biosynthesis, asparagine kinase L-aspartate -> L-asparagine

Two independent mutants in each selected gene were tested for survival over 24 h after exposure to 2X the MIC of aztreonam, ciprofloxacin or gentamicin, selected to represent 3 different classes of bactericidal antibiotics (MICs were based on those against BW25113 and none of the selected mutants demonstrated an elevated MIC).

The results for each mutant were consistent with our hypothesis in showing that nearly all the mutants demonstrated a survival benefit compared to the parent strain (Fig. 3d). There was a high degree of consistency seen between the independent mutants for each gene with mutants in the different genes showing up to 4 log reductions in killing over the time tested. The initial TraDIS experiments had indicated significant log-fold increases in mutations within each of these genes after antibiotic exposure and, whilst the experiments are not like for like, the survival assays support disruption of these pathways as having a relative benefit. Initial survival was typically better for the selected mutants compared to the wild-type, and in ciprofloxacin the mutants were able to restart growth after an extended period (after 6 h). This was again consistent in timing across replicates making selection of secondary mutations unlikely. The benefits of different mutants did vary between the three antibiotics tested although all mutants showed a benefit in some tested conditions.

Together our observations demonstrate that a general reduction in energy generation and precursors for growth confers a selective advantage during exposure to many antibiotics. The phenomenon of antibiotic survival being aided by a general dampening of growth rate ‘tolerance’ has been appreciated as important for some time^2^. Our data here reinforces the likely importance of tolerance as a ‘central’ mechanism by which cells can survive antibiotic exposure in the absence of specific resistance genes and expands understanding of likely pathways within which disruption can provide a survival benefit.

### Aminoacyl-tRNA biosynthesis is important for many antibiotics

**Fig. 4:**
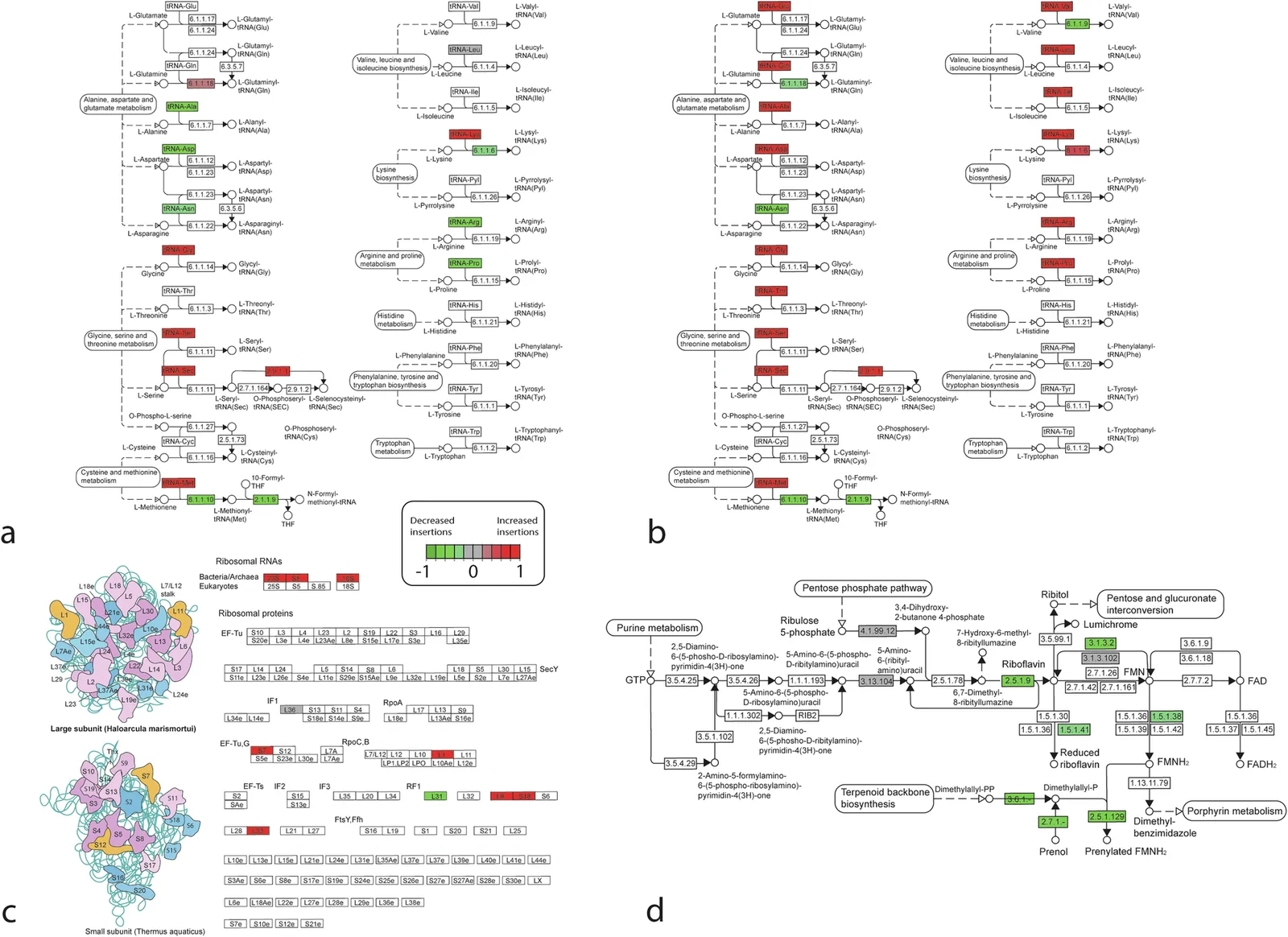
Pathways with conditionally different impacts on survival to different antibiotics. **a**, **b** Show Aminoacyl-tRNA biosynthesis pathways with opposing results after exposure to gentamicin and coumermycin (at 0.25X MIC). **c** Schematic of the ribosome with corresponding genes and significant changes in transposon inserts shown (data after exposure to 1X MIC of gentamicin). **d** Genes within riboflavin metabolism with decreased numbers of inserts after exposure to aztreonam (0.25X MIC, genes with significant differences are shown in green). Pathway illustration adapted by the authors from KEGG pathway information.

Another pathway commonly affected by antibiotic stress was aminoacyl-tRNA biosynthesis. However, the signals seen for this pathway varied between increased importance (fewer insertions) and disruption (more insertions) within the pathway when exposed to different antibiotics. For example, after gentamicin exposure we observed an increase in insertions across many of the pathways producing aminoacyl-tRNAs (Fig. 4a). Gentamicin is an aminoglycoside and acts on the aminoacyl-tRNA site (A site) in the prokaryotic 30S ribosomal subunit so this signal might reflect a specific interaction for this drug^18^.

After exposure to coumermycin, we saw a different pattern, with changes in insertions across different parts of the pathway (Fig. 4b). A similar pattern was observed with several other antibiotics, where parts of or the whole of alanine, aspartate and glutamate metabolism (feeding synthesis of aminoacyl-tRNAs) became more important under exposure to azithromycin, sulfamethoxazole, colistin, fosfomycin, coumermycin and cefuroxime. A common signal of a benefit from disrupting glycine, serine and threonine metabolism was consistently seen for azithromycin, sulfamethoxazole, colistin, coumermycin or cefuroxime.

Methionine biosynthesis showed a specific pattern which was seen in multiple conditions in that tRNA-Met had an increased number of insertions while methionyl-tRNA synthetase and methionyl-tRNA formyltransferase had decreased numbers of insertions. Methionine is particularly important due to its multiple roles in the cell’s physiology and its involvement in numerous cellular processes, including protein synthesis, and methylation of DNA, RNA and proteins^19^. We see the conversion from L-methionine into N-Formyl-methionyl-tRNA, which initiates protein synthesis, becomes more important (depletion of insertion mutants) in all antibiotics demonstrating that maintenance of this function is important for surviving antibiotic exposure. It has been suggested that under certain stress conditions where oxidation is a threat to the cell, higher intracellular levels of methionine are beneficial to maintain normal growth and cellular functions. This is proposed to relate to the high propensity of methionine to be oxidised but without compromising protein function, and the high amount of methionine residues which are surface exposed on some proteins^19,20^. This may explain this consistent signal in our data.

### Riboflavin metabolism and Terpenoid backbone biosynthesis

Riboflavin metabolism exhibited fewer insertions across the pathway following exposure to all tested antibiotics, except for fosfomycin and gentamicin at low concentrations. This suggests that riboflavin metabolism remained largely protected and maintenance of this function is generically protective against antibiotic stress. However, this pathway was generally significant only at the lower antibiotic concentrations, indicating that its protective effects are limited to sub-inhibitory exposure conditions.

Terpenoid scaffold biosynthesis was also important for survival in multiple antibiotics. Terpenoids are a large class of molecules with distinct functions and some members are involved in stress resilience, membrane integrity or redox-balance which may explain their importance in surviving antibiotic exposure.

Figure 4d illustrates this with an example of annotated riboflavin metabolism and terpenoid scaffold biosynthesis pathways, highlighting genes under selective pressure after aztreonam exposure.

### Gentamicin

As previously mentioned, gentamicin showed a distinct pathway signature (Figs. 1, 2). Many of the pathways seen in multiple other drugs were not significant after gentamicin exposure, although disruption of structural ribosomal RNAs, terpenoid biosynthesis, ubiquinones, other terpenoid-quinone biosynthesis, porphyrin metabolism, RNA degradation and biosynthesis of cofactors appeared beneficial. This signature is contrary to the signal in most other antibiotics examined. Figure 4c shows the ribosomal RNAs had increased transposon insertions after gentamicin exposure. It has previously been reported that mutations within the different regions of the ribosomal RNAs can confer resistance to several aminoglycosides^21^ which is consistent to this observation and we also saw similar data for the beta-lactams (for example cefuroxime) although how this mechanistically relates is unclear.

### Overexpression of some pathways was beneficial to survival

**Fig. 5:**
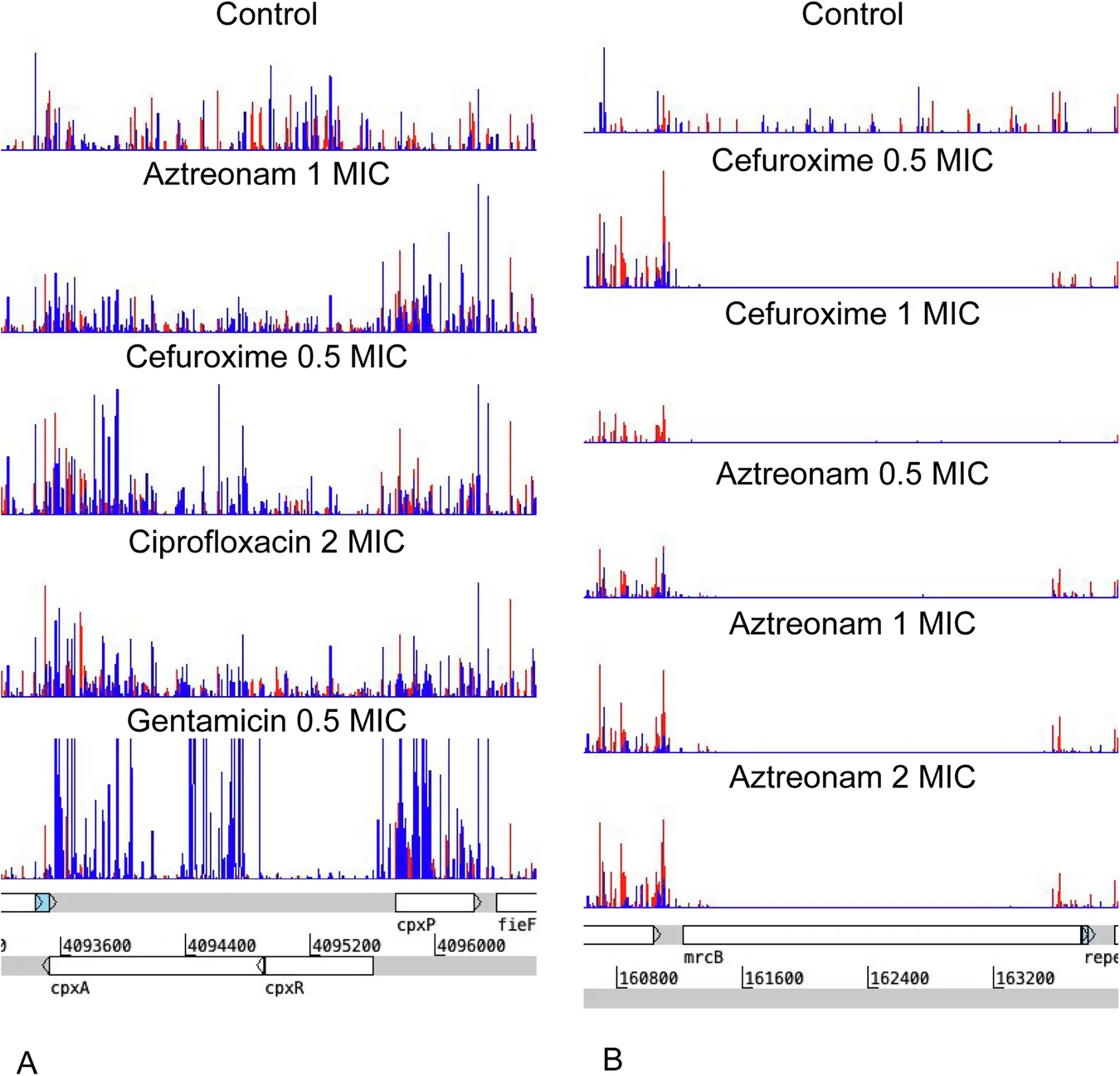
Examples where expression changes impacted susceptibility. Genes are indicated at the bottom and mutant abundance shown above by vertical lines the height of which is proportional to the number of insertion mutants at that site; red indicates transposon insertions oriented to transcribe to the right and blue to the left. **A** Insertion mutations within and flanking *cpxR* after exposure to different concentrations of aztreonam, cefuroxime, ciprofloxacin, and gentamicin select for mutations that inactivate *cpxP* and increase expression of *cpxR*. **B** Mutations within *mrcB* are depleted, and those 5’ to the gene that increase expression have enriched after exposure to aztreonam and cefuroxime. This figure was generated using Artemis genome browser output using study data.

Analysis of pathway enrichment allows us to identify major cellular events which impact antibiotic susceptibility. TraDIS-*Xpress* also has the advantage over other TnSeq based screens of using an outward facing promoter within the transposons so we can identify where expression changes to individual genes impact fitness in the presence of antibiotics. We observed a series of loci where over-expression of a gene was predicted to be highly beneficial (even if many mutants within the total sample had died), for example, Fig. 5A shows both protection of, and enrichment of increased expression mutants for *mcrB* after exposure to the beta-lactam antibiotics aztreonam or cefuroxime. The major target of aztreonam and cefuroxime is penicillin binding protein (PBP)3, a transpeptidase (peptide cross-linking enzyme) involved in septal peptidoglycan synthesis encoded by *ftsI*. The *mrcB* gene codes for PBP1b, a peptidoglycan synthase that possesses both glycosyltransferase (glycan chain polymerising) and transpeptidase activities. Our data indicate that disruption to the FtsI (PBP3) transpeptidase can be countered by increasing transpeptidase activity of MrcB (PBP1b).

Another example where over-expression of a gene was beneficial was *cpxR*. As with *mrcB*, this was seen after exposure to aztreonam or cefuroxime, but also with unrelated drugs, ciprofloxacin and gentamicin. These drugs were selected for both reduced insertions within the gene and an increase in transcriptional activating insertions located 5’ (Fig. 5B). CpxR is part of the CpxA-CpxR two-component system, a key regulatory system that responds to extracytoplasmic stress^22,23^. In this system, CpxA is a sensor kinase, and CpxR is a response regulator. Only *cpxR* was identified as being under selection rather than the cognate partner, *cpxA*. This suggests that regulatory functions of CpxR are key to the phenotypic benefits of this system under antibiotic stress. CpxRA has been shown to be involved in the response to agents which can harm the bacterial envelope^24^ and can alter transcription of genes involved in protein folding and degradation, and cell envelope repair, both pathways linked to antibiotic susceptibility^25^. Consistent with this, recent work has demonstrated that activation of envelope stress responses, including Cpx, promotes survival under β-lactam exposure by enabling repair of antibiotic-induced damage^25,26^. CpxR can also regulate the expression of multidrug efflux pumps including AcrAB-TolC^25–28^ which is well recognised as being able to export most classes of antibiotics from the cell.

### Transport

Whilst alterations to central metabolic pathways will have detrimental impacts on energy production and growth rate, disruption of the electron transport chain also impacts secondary active transport as the proton gradient is also used to power the transport of various molecules across the cell membrane. This is the major power source for multidrug efflux pumps in *E. coli* which can export a wide variety of antibiotics. Signals relating to the importance of transport were seen although these varied between specific antibiotics. We saw various cases where there was a predicted benefit from the disruption of an importer, highlighting routes of antibiotic entry although these were often drug specific (e.g. GlpT and UhpT are required for fosfomycin entry and disruption of these is enriched after fosfomycin exposure). More consistent signals were seen for some multidrug efflux exporters for example insertion mutants for AcrAB, MacB, which were generally depleted after exposure to many of the agents tested reflecting their known roles in export of multiple antibiotics^29^.

## Discussion

Using a TraDIS-Xpress transposon mutant library in *E. coli* K-12 combined with pathway enrichment analysis, we systematically linked genotypic and phenotypic responses to 13 antibiotics representing all major classes. This genome-wide approach enables parallel assessment of the contribution of all genes, including non-coding regions, to survival under antibiotic stress. The inclusion of an outward-transcribing promoter within the transposon not only allowed the effects of gene inactivation to be examined, but also of altered gene expression. Together, these data reveal a broad landscape of genetic determinants influencing antibiotic survival, encompassing both drug-specific interactions and conserved physiological responses.

Although no single gene or pathway was required for survival across all antibiotics tested, we observed a strong and consistent signal of selection for disruption of non-target pathways, particularly those involved in metabolism, growth, and amino acid biosynthesis.

Experimental validation of selected mutants confirmed that loss of function in key metabolic and respiratory pathways significantly enhances survival across multiple antibiotic classes. These findings reinforce the concept that modulation of central cellular physiology, rather than alteration of drug targets alone can have important impacts on antibiotic susceptibility. We extend the pathways known to contribute to antibiotic susceptibility by comparing the selective importance of all in parallel. Most of these pathways are likely to contribute to antibiotic tolerance rather than resistance and were consistently under positive selection from lower concentrations of antibiotics in our experiments. The connection between antibiotic tolerance and bacterial metabolism has been noted previously^5,12^, and several studies have proposed strategies to reverse tolerance by stimulating metabolic activity^30^. For example, supplementation with exogenous metabolites such as amino acids, TCA cycle intermediates, or nucleotides has been shown to restore antibiotic sensitivity in tolerant populations. Addition of specific amino acids, including L-alanine, can resensitise bacteria to gentamicin by activating the TCA cycle^30^, while other studies have demonstrated that glycine, serine, threonine, or glucose can enhance antibiotic killing across different species^31^. Our genome-scale data provide a systematic genetic basis to provide mechanistic support for these observations, identifying the metabolic pathways whose disruption promotes survival and whose reactivation may therefore represent a route to therapeutic intervention.

By defining the genome-wide architecture of antibiotic susceptibility, this work identifies multiple pathways which assist survival under antibiotic stress. Changes to these pathways which enable bacterial populations to survive antibiotic exposure may increase opportunities for the further acquisition and fixation of resistance-conferring mutations. The pathways identified here therefore represent genomic features that are likely to shape evolutionary trajectories toward antimicrobial resistance and contribute to treatment failure. This improved understanding of the landscape of susceptibility associated genes and pathways may improve our ability to predict antibiotic outcomes and identify vulnerabilities that can be exploited to limit resistance evolution, or to counter resistance after it has evolved. This study focuses on one species, and one strain. Whilst many of the pathways we identify are part of the core genome shared across Enterobacteriaceae further work should explore other strains and species to identify how different bacteria have evolved to deal with antimicrobial stress. More broadly, the conceptual and methodological framework established here can be applied to other Gram-negative pathogens to uncover common principles governing antibiotic survival and antimicrobial resistance.

## Methods

### TraDIS-Xpress transposon mutant library

Construction of the high-density TraDIS-*Xpress* transposon mutant library (containing approximately 800,000 different insertion mutants with one insertion on average every 6 bp across the genome) in *E. coli* BW25113 has been described^16,32^. BW25113 was chosen as this strain has been a standard reference for understanding the genome of *E. coli* with a comprehensively annotated genome nucleotide sequence and it is the parent strain for the KEIO collection of defined mutants^33^. The transposon used is a mini-Tn5 derivative coding for kanamycin resistance [*aph(3')-Ia*] and incorporates an outward-transcribing *tac* promoter 3′ of the kanamycin resistance gene that is inducible by IPTG, allowing over-expression of genes adjacent to transposon insertions into which the transposon *tac* promoter transcribes. Similarly, transposon insertions at the ends of genes, that transcribe into the gene in the reverse direction, can suppress gene expression due to the synthesis of a reverse complementary RNA transcript. This allows genes to be assayed by altered expression, including essential genes which cannot be assayed by insertional inactivation as these mutations are lethal and such mutants are therefore absent from the transposon mutant library.

### Antibiotic exposure conditions and TraDIS-Xpress sequencing

We exposed the mutant library to a panel of 13 antibiotics selected to include major clinically relevant classes and representatives within these as well as drugs with different primary modes of action. The library was exposed to each drug at 0.25X, 0.5X, 1X and 2X the corresponding MIC against BW25113 (determined using microbroth dilution in LB broth; Supplementary Table 1) to allow assessment of responses to sub and super inhibitory concentrations of each drug.

For TraDIS-*Xpress* experiments, approximately 10^−7^ mutants were inoculated into 1.7 mL of LB broth in deep 96-well plates containing antibiotics (and an antibiotic-free control). Replicate experiments were completed with no induction, or with the addition of 0.2 or 1 mM IPTG to induce transcription from the transposon outward-transcribing *tac* promoter. Mutants were grown for 18 h at 37 °C before DNA was harvested from each experiment. All experiments were performed in duplicate to give a total of 30 independent TraDIS-*Xpress* experiments (Fig. 1).

After growth, DNA was extracted from each experiment using a Quick-DNA™ Fungal/Bacterial 96 Kit (Zymo Research) before being fragmented with a Nextera DNA library preparation kit (Illumina) although Tnp-i5 oligonucleotides replaced i5 index primers, and 28 PCR cycles were used. This DNA was then sequenced on a NEXTSeq 500 sequencing machine using a NextSeq 500/550 High Output v2 kit (75 cycles). All sequence data have been deposited with NCBI under project accession number PRJNA1473163.

### Bioinformatics

Results were analysed using BioTraDIS (version 1.4.1)^34^ and AlbaTraDIS (version 0.0.5)^35^, specifically developed for TraDIS-*Xpress* analysis^16,32^. Sequencing reads from (-IPTG) and (+IPTG) samples were pooled for all downstream analyses. BioTraDIS matched sequence reads against the reference genome (CP009273) using SMALT^36^ and created insertion plots of mapped transposon insertion sites. Reads numbers were normalised across samples to allow comparison. AlbaTraDIS compared the number of inserts within each gene between conditions and identified significant differences (each test condition compared to drug-free controls) on a per-gene basis using a negative binomial distribution and an adapted exact test as implemented in edgeR^37^ followed by multiple testing correction^38^. The insertion patterns at candidate loci identified as important were also visually inspected using the Artemis genome browser, which was also used to capture images for figures^39^.

### Gene enrichment and pathway enrichment analysis

Gene set enrichment analysis (GSEA) was performed to identify coordinated effects across groups of genes associated with shared biological functions or pathways. Differential insertion analysis was first conducted using edgeR for each condition (antibiotic and concentration) relative to the corresponding control. Genes were ranked based on the signed log2 fold change (logFC) of insertion frequency, preserving the directionality of selection.

Enrichment analysis was carried out using the R package *clusterProfiler*^40^, applying the gseKEGG function with the KEGG pathway database^41^. Default parameters were used unless otherwise stated, with 10,000 permutations to estimate statistical significance. Enrichment scores (ES), normalised enrichment scores (NES), and leading-edge subsets were calculated for each pathway. Positive NES values indicate enrichment of genes with increased insertion frequency (suggesting a selective advantage), while negative NES values indicate depletion of insertions (suggesting a selective disadvantage). Pathways were considered significant at a nominal p-value of <0.05 and interpreted conservatively in the context of consistency across conditions and biological plausibility.

Principal component analysis (PCA) was performed in R using the matrix of NES values across all conditions and replicates to assess reproducibility and global similarity between conditions (Fig. 1). Heatmaps were generated using the same NES matrix to visualise enrichment patterns across conditions (Fig. 2).

Individual KEGG pathways were visualised using the *pathview* package^42^ with logFC values mapped onto pathway diagrams. For multi-gene complexes, values were aggregated to represent the overall direction of change, scaled between −1 and 1 for visualisation purposes.

### Validation experiments

A series of pathways were identified as being important across multiple antibiotics including glycine, serine and threonine metabolism, the TCA cycle, oxidative phosphorylation, and aminoacyl t-RNA biosynthesis. Defined inactivation mutants from the Keio collection^33^ were selected to represent loss of function of these pathways and then used to test the predicted impacts on antimicrobial susceptibility from the TraDIS-*Xpress* dataset (Table 1).

The Keio collection mutant library contains two independent insertion knockout mutants for each gene, and both replicates were used, as well as two biological replicates of wild-type *E. coli* BW25113 as a parent control. Each mutant was exposed to three representative bactericidal antibiotics, aztreonam (a beta-lactam, acting on cell wall), ciprofloxacin (acting on DNA housekeeping) and gentamicin (acting on the ribosome). The defined mutants and wild-type *E. coli* control were resuscitated from glycerol stocks by inoculation of 1 µl into 5 ml of LB broth before overnight incubation (~24 h) at 37 °C and 200 rpm. The optical density of each culture was normalised to 0.7 (measured at 600 nm) with pre-warmed LB broth. Mutants and wild-type *E. coli* were then exposed to double the minimum inhibitory concentration of aztreonam, ciprofloxacin and gentamicin. Cell numbers at 0 h and 2, 4 and 6 h after exposure to each antibiotic were determined using the Miles and Misra method of calculating colony forming units (Miles, Misra, and Irwin 1938).

## Supplementary information

NPJ_SUP

## Data Availability

All sequence data have been deposited with NCBI under project accession number PRJNA1473163.

## Code availability

All analysis code is publicly available on GitHub (quadram-institute-bioscience/QuaTradis and quadram-institute-bioscience/TraDIS) under an open-source license.

## References

[1] Westblade, L. F., Errington, J. & Dörr, T. Antibiotic tolerance. *PLOS P athogens* **16**, e1008892 (2020). 10.1371/journal.ppat.1008892 PMC7561168 33057409

[2] Balaban, N. Q. et al. Definitions and guidelines for research on antibiotic persistence. *Nat. Rev. Microbiol.* **17**, 441–448 (2019). 10.1038/s41579-019-0196-3 PMC7136161 30980069

[3] Darby, E. M. et al. Molecular mechanisms of antibiotic resistance revisited. *Nat. Rev. Microbiol.* **21**, 280–295 (2023). 10.1038/s41579-022-00820-y 36411397

[4] Meléndez-Carmona, M. Á., Muñoz-Gallego, I., Mancheño-Losa, M. & Lora-Tamayo, J. A Study of Antibiotic Tolerance to Levofloxacin and Rifampin in isolates causing prosthetic joint infections: clinical relevance and treatment challenges. *Antibiotics* **15**, 10 (2025). 10.3390/antibiotics15010010 PMC12837759 41594048

[5] Brauner, A., Fridman, O., Gefen, O. & Balaban, N. Q. Distinguishing between resistance, tolerance and persistence to antibiotic treatment. *Nat. Rev. Microbiol.* **14**, 320–330 (2016). 10.1038/nrmicro.2016.34 27080241

[6] Wood, T. K., Knabel, S. J. & Kwan, B. W. Bacterial persister cell formation and dormancy. *Appl Environ. Microbiol.* **79**, 7116–7121 (2013). 10.1128/AEM.02636-13 PMC3837759 24038684

[7] Jana, B. et al. The secondary resistome of multidrug-resistant Klebsiella pneumoniae. *Sci. Rep.* **7**, 42483 (2017). 10.1038/srep42483 PMC5309761 28198411

[8] Santiago, M. et al. Genome-wide mutant profiling predicts the mechanism of a Lipid II binding antibiotic. *Nat. Chem. Biol.* **14**, 601–608 (2018). 10.1038/s41589-018-0041-4 PMC5964011 29662210

[9] Ward, R. D. et al. Essential gene knockdowns reveal genetic vulnerabilities and antibiotic sensitivities in. *mBio* **15**, e0205123 (2024). 10.1128/mbio.02051-23 PMC10865783 38126769

[10] Gilmore, M. S. et al. Genes contributing to the unique biology and intrinsic antibiotic resistance of Enterococcus faecalis. *mBio* **11**, 10–1128 (2020). 10.1128/mBio.02962-20 PMC7701990 33234689

[11] Leshchiner, D. et al. A genome-wide atlas of antibiotic susceptibility targets and pathways to tolerance. *Nat. Commun.* **13**, 3165 (2022). 10.1038/s41467-022-30967-4 PMC9174251 35672367

[12] Shan, Y., Lazinski, D., Rowe, S., Camilli, A. & Lewis, K. Genetic basis of persister tolerance to aminoglycosides in *Escherichia coli*. *mBio* **6**, 10–1128 (2015). 10.1128/mBio.00078-15 PMC4453570 25852159

[13] Marciano, D. C. et al. Evolutionary action of mutations reveals antimicrobial resistance genes in Escherichia coli. *Nat. Commun.* **13**, 3189 (2022). 10.1038/s41467-022-30889-1 PMC9184624 35680894

[14] Roemhild, R., Bollenbach, T. & Andersson, D. I. The physiology and genetics of bacterial responses to antibiotic combinations. *Nat. Rev. Microbiol.* **20**, 478–490 (2022). 10.1038/s41579-022-00700-5 35241807

[15] Langridge, G. C. et al. Simultaneous assay of every Salmonella Typhi gene using one million transposon mutants. *Genome Res.* **19**, 2308–2316 (2009). 10.1101/gr.097097.109 PMC2792183 19826075

[16] Yasir, M. et al. TraDIS-Xpress: a high-resolution whole-genome assay identifies novel mechanisms of triclosan action and resistance. *Genome Res.* **30**, 239–249 (2020). 10.1101/gr.254391.119 PMC7050523 32051187

[17] Erhardt, H. et al. Disruption of individual nuo-genes leads to the formation of partially assembled NADH:ubiquinone oxidoreductase (complex I) in Escherichia coli. *Biochim. Biophys. Acta* **1817**, 863–871 (2012). 10.1016/j.bbabio.2011.10.008 22063474

[18] Tsai, A. et al. The impact of aminoglycosides on the dynamics of translation elongation. *Cell Rep.* **3**, 497–508 (2013). 10.1016/j.celrep.2013.01.027 PMC3766726 23416053

[19] Jochim, A. et al. Methionine limitation impairs pathogen expansion and biofilm formation capacity. *Appl. Environ. Microbiol.* **85**, e00177–19 (2019). 10.1128/AEM.00177-19 PMC6495772 30824455

[20] Singh, V. K., Singh, K. & Baum, K. The role of methionine sulfoxide reductases in oxidative stress tolerance and virulence of and other bacteria. *Antioxidants* **7**, 128 (2018). 10.3390/antiox7100128 PMC6210949 30274148

[21] De Stasio, E. A., Moazed, D., Noller, H. F. & Dahlberg, A. E. Mutations in 16S ribosomal RNA disrupt antibiotic-RNA interactions. *EMBO J.* **8**, 1213–1216 (1989). 10.1002/j.1460-2075.1989.tb03494.x PMC400937 2472961

[22] Miles, A. A., Misra, S. S. & Irwin, J. O. The estimation of the bactericidal power of the blood. *Epidemiol. Infect.* **38**, 732–749 (1938). 10.1017/s002217240001158x PMC2199673 20475467

[23] Hunke, S., Keller, R. & Müller, V. S. Signal integration by the Cpx-envelope stress system. *FEMS Microbiol. Lett.* **326**, 12–22 (2012). 10.1111/j.1574-6968.2011.02436.x 22092888

[24] Delhaye, A., Collet, J.-F. & Laloux, G. A Fly On The Wall: How Stress Response Systems Can Sense And Respond To Damage To Peptidoglycan. *Front. Cell Infect. Microbiol.* **9**, 380 (2019). 10.3389/fcimb.2019.00380 PMC6863773 31799211

[25] Hirakawa, H., Nishino, K., Yamada, J., Hirata, T. & Yamaguchi, A. Beta-lactam resistance modulated by the overexpression of response regulators of two-component signal transduction systems in *Escherichia coli*. *J. Antimicrob. Chemother.* **52**, 576–582 (2003). 10.1093/jac/dkg406 12951338

[26] Cross, T. et al. Prevalence and mechanisms of high-level carbapenem antibiotic tolerance in clinical isolates of Klebsiella pneumoniae. *PLoS Pathog.* **22**, e1013919 (2026). 10.1371/journal.ppat.1013919 PMC12880740 41616033

[27] Du, D. et al. Multidrug efflux pumps: structure, function and regulation. *Nat. Rev. Microbiol* **16**, 523–539 (2018). 10.1038/s41579-018-0048-6 30002505

[28] Nishino, K. & Yamaguchi, A. Analysis of a complete library of putative drug transporter genes in *Escherichia coli*. *J. Bacteriol.* **183**, 5803–5812 (2001). 10.1128/JB.183.20.5803-5812.2001 PMC99656 11566977

[29] Greene, N. P., Kaplan, E., Crow, A. & Koronakis, V. Antibiotic Resistance Mediated by the MacB ABC transporter family: a structural and functional perspective. *Front. Microbiol.* **9**, 950 (2018). 10.3389/fmicb.2018.00950 PMC5985334 29892271

[30] Liu, Y., Yang, K., Zhang, H., Jia, Y. & Wang, Z. Combating antibiotic tolerance through activating bacterial metabolism. *Front. Microbiol.* **11**, 577564 (2020). 10.3389/fmicb.2020.577564 PMC7642520 33193198

[31] Cheng, Z.-X. et al. Glycine, serine and threonine metabolism confounds efficacy of complement-mediated killing. *Nat. Commun.* **10**, 3325 (2019). 10.1038/s41467-019-11129-5 PMC6658569 31346171

[32] Turner, A. K. et al. A genome-wide analysis of *Escherichia coli* responses to fosfomycin using TraDIS-Xpress reveals novel roles for phosphonate degradation and phosphate transport systems. *J. Antimicrob. Chemother.* **75**, 3144–3151 (2020). 10.1093/jac/dkaa296 PMC7566553 32756955

[33] Baba, T. et al. Construction of *Escherichia coli* K-12 in-frame, single-gene knockout mutants: the Keio collection. *Mol. Syst. Biol.* **2**, 2006.0008 (2006). 10.1038/msb4100050 PMC1681482 16738554

[34] Barquist, L. et al. The TraDIS toolkit: sequencing and analysis for dense transposon mutant libraries. *Bioinformatics* **32**, 1109–1111 (2016). 10.1093/bioinformatics/btw022 PMC4896371 26794317

[35] Page, A. J. et al. AlbaTraDIS: comparative analysis of large datasets from parallel transposon mutagenesis experiments. *PLoS Comput. Biol.* **16**, e1007980 (2020). 10.1371/journal.pcbi.1007980 PMC7390408 32678849

[36] Website. Wellcome Sanger Institute. 2011. SMALT: A Mapper for DNA Sequencing Reads. Available at: https://www.sanger.ac.uk/tool/smalt/.

[37] Robinson, M. D., McCarthy, D. J. & Smyth, G. K. edgeR: a Bioconductor package for differential expression analysis of digital gene expression data. *Bioinformatics* **26**, 139–140 (2010). 10.1093/bioinformatics/btp616 PMC2796818 19910308

[38] Japkowicz, N. & Stefanowski, J. *Big Data Analysis: New Algorithms for a New Society* (Springer International Publishing, 2015).

[39] Carver, T., Harris, S. R., Berriman, M., Parkhill, J. & McQuillan, J. A. Artemis: an integrated platform for visualization and analysis of high-throughput sequence-based experimental data. *Bioinformatics* **28**, 464–469 (2012). 10.1093/bioinformatics/btr703 PMC3278759 22199388

[40] Wu, T. et al. clusterProfiler 4.0: a universal enrichment tool for interpreting omics data. *Innov. (Camb.)* **2**, 100141 (2021). 10.1016/j.xinn.2021.100141 PMC8454663 34557778

[41] Kanehisa, M. & Goto, S. KEGG: kyoto encyclopedia of genes and genomes. *Nucleic Acids Res.* **28**, 27–30 (2000). 10.1093/nar/28.1.27 PMC102409 10592173

[42] Luo, W. & Brouwer, C. Pathview: an R/Bioconductor package for pathway-based data integration and visualization. *Bioinformatics* **29**, 1830–1831 (2013). 10.1093/bioinformatics/btt285 PMC3702256 23740750

