# Supplementary material for: Mapping the genomic landscape of antibiotic susceptiblity in Escherichia coli
Source: NPJ Antimicrob Resist. 2026 Jun 4;4:63. doi: 10.1038/s44259-026-00227-2 (PMC13645799; doi:10.1038/s44259-026-00227-2)
Supplement: Supplementary file 1 [file 44259_2026_227_MOESM1_ESM.docx]

**Supporting Information for**

**Mapping the landscape of antibiotic tolerance in *Escherichia coli***

Sarah Bastkowski*^1,2^, Keith Turner^1,2^, Muhammad Yasir^1,2^, Caroline Jarvis^1,2,3^, Ian Charles^1,2,3^ , Mark Webber^1,2,3*^

^1^Quadram Institute Bioscience, Norwich Research Park, Norwich, NR4 7UQ, UK

^2^Centre for Microbial Interactions, Norwich Research Park, Norwich, NR4 7TJ, UK

^3^Norwich Medical School, University of East Anglia, Norwich, NR4 7TJ, UK

Sarah Bastkowski

**Email:**  [sarah.bastkowski@quadram.ac.uk](mailto:sarah.bastkowski@quadram.ac.uk)

**Supplementary figure 1: Consistency of responses between replicates.**

PCA plot based on the sample-wise vector of insertion indexes per gene. Separate replicates are denoted by ‘1’ or ‘2’ after the relevant antibiotic name and followed by the exposure concentration.

**Supplementary figure 2. Example of degradation of the *nuo* operon after exposure to gentamicin.**

This figure shows the transposon insertion pattern in the nuo genes for different Gentamycin concentrations compared to the controls. Genes are indicated at the bottom and mutant abundance shown above by vertical lines the height of which is proportional to the number of mutants; red indicates transposon insertions oriented to transcribe to the right and blue to the left.

**Supplementary table 1. Antibiotics tested in this study.**

| Antibiotic | Mode of action | Effect | MIC |
| --- | --- | --- | --- |
| Azithromycin | Ribosome | bacteriostatic | 32 µg/ml |
| Aztreonam | Cell Wall | bactericidal | 0.0625 µg/ml |
| Cefuroxime | Cell Wall | bactericidal | 8 µg/ml |
| Ciprofloxacin | DNA supercoiling | bactericidal | 0.03125 µg/ml |
| Coumermycin | DNA supercoiling | bactericidal | 16 µg/ml |
| Gentamicin | Ribosome | bactericidal | 16 µg/ml |
| Meropenem | Cell Wall | bactericidal | 0.032 µg/ml |
| Piperacillin | Cell Wall | bactericidal | 4 µg/ml |
| Tigecycline | Ribosome | bacteriostatic | 1 µg/ml |
| Fosfomycin | Cell Wall | bactericidal | 1 µg/ml |
| Sulfamethoxazole | DNA synthesis | bacteriostatic | 2048 µg/ml |
| Trimethoprim | DNA synthesis | bacteriostatic | 1 µg/ml |
| Colistin | Cell membrane | bactericidal | 0.5 µg/ml |

MIC shown relates to BW25113 in LB broth.

**Supplementary table 2: Number of reads, reads matched with tag and then aligned to the genome as well as unique insertion sites.**

| Sample | Total Reads | Reads Matched | Reads Mapped | Total Unique Insertion Sites |
| --- | --- | --- | --- | --- |
| Azithromycin rep1 0.25 MIC | 29604876 | 17174100 | 12021291 | 1713344 |
| Azithromycin rep1 2 MIC | 19208776 | 10920699 | 7787320 | 1885386 |
| Azithromycin rep1 0.5 MIC | 22979588 | 15478270 | 11443195 | 2127017 |
| Azithromycin rep1 1 MIC | 31080878 | 19893462 | 15322015 | 2328079 |
| Azithromycin rep20.25 MIC | 27724816 | 19026859 | 14380337 | 2432186 |
| Azithromycin rep2 2 MIC | 35278643 | 21878448 | 16294167 | 2163954 |
| Azithromycin rep2 0.5 MIC | 30845383 | 19799269 | 14395361 | 2190501 |
| Azithromycin rep2 1 MIC | 25379171 | 17426808 | 13339855 | 2116807 |
| Aztreonam rep1 0.25 MIC | 30557180 | 18294744 | 12945451 | 1868003 |
| Aztreonam rep1 2 MIC | 12809541 | 7535459 | 4554068 | 1397465 |
| Aztreonam rep1 0.5 MIC | 14759608 | 9841141 | 6557781 | 1514040 |
| Aztreonam rep1 1 MIC | 28051393 | 17581486 | 9985223 | 1766216 |
| Aztreonam rep2 0.25 MIC | 32452768 | 21258305 | 15544741 | 2088601 |
| Aztreonam rep2 2 MIC | 25352416 | 16574937 | 10490637 | 1931085 |
| Aztreonam rep2 0.5 MIC | 23997269 | 15374244 | 9140988 | 1835657 |
| Aztreonam rep2 1 MIC | 18485558 | 12232714 | 6539456 | 1316010 |
| Cefuroxime rep1 0.25 MIC | 32722161 | 20704960 | 15436516 | 932398 |
| Cefuroxime rep1 2 MIC | 31179634 | 20483832 | 15635821 | 667192 |
| Cefuroxime rep1 0.5 MIC | 15420228 | 9890062 | 7544441 | 353300 |
| Cefuroxime rep1 1 MIC | NA | NA | NA | NA |
| Cefuroxime rep2 0.25 MIC | 20504071 | 13654959 | 10384028 | 1522054 |
| Cefuroxime rep2 2 MIC | 18736465 | 13125597 | 10842361 | 39759 |
| Cefuroxime rep2 0.5 MIC | 22195460 | 15087846 | 10851801 | 1617785 |
| Cefuroxime rep2 1 MIC | 19440393 | 12602828 | 9639180 | 176510 |
| Ciprofloxacin rep1 0.25 MIC | 16770359 | 9816420 | 6692874 | 1237692 |
| Ciprofloxacin rep1 2 MIC | 23896199 | 13905313 | 9404834 | 1370240 |
| Ciprofloxacin rep1 0.5 MIC | 22261126 | 13499067 | 9371239 | 1338319 |
| Ciprofloxacin rep1 1 MIC | 23568076 | 14292087 | 9662650 | 1504185 |
| Ciprofloxacin rep2 0.25 MIC | 39447870 | 24532807 | 16884576 | 1665555 |
| Ciprofloxacin rep2 2 MIC | 31299613 | 20656188 | 14980531 | 1615425 |
| Ciprofloxacin rep2 0.5 MIC | 19478170 | 12520354 | 8741873 | 1532856 |
| Ciprofloxacin rep2 1 MIC | 26075441 | 15810978 | 10831937 | 1860555 |
| Coumermycin rep1 0.25 MIC | 28548233 | 19282758 | 13451529 | 1669554 |
| Coumermycin rep1 2 MIC | 23040112 | 16342951 | 12575216 | 878908 |
| Coumermycin rep1 0.5 MIC | 32505025 | 22898308 | 16906006 | 2014105 |
| Coumermycin rep1 1 MIC | 25456654 | 17525894 | 13352619 | 1550549 |
| Coumermycin rep2 0.25 MIC | 28560610 | 20540981 | 15830175 | 2007840 |
| Coumermycin rep2 2 MIC | 23603507 | 17829207 | 14841310 | 933597 |
| Coumermycin rep2 0.5 MIC | 30374757 | 21003521 | 16036056 | 2136765 |
| Coumermycin rep2 1 MIC | 27883818 | 20099646 | 16104231 | 2259627 |
| Gentamicin rep1 0.25 MIC | 27291385 | 15616223 | 10444773 | 1671664 |
| Gentamicin rep1 2 MIC | 37076512 | 18393898 | 11323438 | 743457 |
| Gentamicin rep1 0.5 MIC | 25585555 | 17225739 | 10218474 | 752883 |
| Gentamicin rep1 1 MIC | 26476457 | 18161775 | 12957614 | 1134608 |
| Gentamicin rep2 0.25 MIC | 29794894 | 20123309 | 14454284 | 2047495 |
| Gentamicin rep2 2 MIC | 29891601 | 15799061 | 9857866 | 767983 |
| Gentamicin rep2 0.5 MIC | 37967054 | 24826668 | 14155125 | 893946 |
| Gentamicin rep2 1 MIC | 15424423 | 11474845 | 8597013 | 1048793 |
| Meropenem rep1 0.25 MIC | 34874655 | 20298877 | 13546500 | 1753358 |
| Meropenem rep1 2 MIC | 21604345 | 12296914 | 7860910 | 1507892 |
| Meropenem rep1 0.5 MIC | 16486991 | 10524437 | 7037728 | 1459145 |
| Meropenem rep1 1 MIC | 56483251 | 33781496 | 18796428 | 1756295 |
| Meropenem rep2 0.25 MIC | 38835664 | 24677958 | 17460147 | 2175113 |
| Meropenem rep2 2 MIC | 26997022 | 16989240 | 11186556 | 1717846 |
| Meropenem rep2 0.5 MIC | 31702362 | 19121997 | 12401189 | 1866274 |
| Meropenem rep2 1 MIC | 24471132 | 15993591 | 9429926 | 1552422 |
| Piperacillin rep1 0.25 MIC | 41219649 | 25253233 | 20076349 | 2383702 |
| Piperacillin rep1 2 MIC | 15278947 | 8690677 | 6141757 | 1477976 |
| Piperacillin rep1 0.5 MIC | 21353197 | 13821487 | 10623160 | 1804246 |
| Piperacillin rep1 1 MIC | 12821555 | 6593943 | 4448525 | 1095168 |
| Piperacillin rep2 0.25 MIC | 22842498 | 13732221 | 10030406 | 1700930 |
| Piperacillin rep2 2 MIC | 24351470 | 14546452 | 10504337 | 1812370 |
| Piperacillin rep2 0.5 MIC | 46638441 | 30065978 | 22662442 | 2381497 |
| Piperacillin rep2 1 MIC | 18247106 | 10365697 | 7336272 | 1566265 |
| Tigecycline rep1 0.25 MIC | 28136158 | 15384166 | 11248287 | 1551103 |
| Tigecycline rep1 2 MIC | 15163170 | 6148750 | 3529256 | 558460 |
| Tigecycline rep1 0.5 MIC | 32601716 | 17399907 | 12353494 | 1148017 |
| Tigecycline rep1 1 MIC | 18027523 | 10780571 | 8111071 | 1584993 |
| Tigecycline rep2 0.25 MIC | 10124467 | 4089095 | 2173542 | 419256 |
| Tigecycline rep2 2 MIC | 9504318 | 4770896 | 3045229 | 916493 |
| Tigecycline rep2 0.5 MIC | 11385936 | 6065879 | 4055499 | 868528 |
| Tigecycline rep2 1 MIC | 8567326 | 4798788 | 3227314 | 1105194 |
| Fosfomycin rep1 0.25 MIC | 41288314 | 29307985 | 22301611 | 2373005 |
| Fosfomycin rep1 2 MIC | 20740967 | 14907067 | 12333896 | 46044 |
| Fosfomycin rep1 0.5 MIC | 29290518 | 21403404 | 17173517 | 188760 |
| Fosfomycin rep1 1 MIC | 14849031 | 11027551 | 9206302 | 67776 |
| Fosfomycin rep2 0.25 MIC | 28700375 | 18863617 | 13265671 | 2151561 |
| Fosfomycin rep2 2 MIC | 20921221 | 14514645 | 11188788 | 65523 |
| Fosfomycin rep2 0.5 MIC | 21398953 | 13703083 | 9816126 | 283511 |
| Fosfomycin rep2 1 MIC | 16607835 | 11439156 | 8614595 | 99490 |
| Sulfamethoxazole rep1 0.25 MIC | 31573194 | 19701737 | 15175644 | 1971268 |
| Sulfamethoxazole rep1 2 MIC | 19025000 | 10272954 | 7490216 | 1515818 |
| Sulfamethoxazole rep1 0.5 MIC | 10393317 | 5772926 | 3287841 | 275045 |
| Sulfamethoxazole rep1 1 MIC | 21781307 | 13028304 | 9819519 | 1686460 |
| Sulfamethoxazole rep2 0.25 MIC | 22326915 | 11833548 | 7978564 | 1285951 |
| Sulfamethoxazole rep2 2 MIC | 28528481 | 16878062 | 12103707 | 1550047 |
| Sulfamethoxazole rep2 0.5 MIC | 34761240 | 19265917 | 13578826 | 1555430 |
| Sulfamethoxazole rep2 1 MIC | 18637162 | 7486886 | 3904021 | 503697 |
| Trimethoprim rep1 0.25 MIC | 13182350 | 7671157 | 5186993 | 1214814 |
| Trimethoprim rep1 2 MIC | 9305339 | 5620022 | 3899445 | 1072039 |
| Trimethoprim rep1 0.5 MIC | 20738074 | 12429834 | 8726040 | 1423081 |
| Trimethoprim rep1 1 MIC | 16066815 | 9613747 | 6442110 | 1427010 |
| Trimethoprim rep2 0.25 MIC | 47122909 | 29855167 | 20432202 | 2213928 |
| Trimethoprim rep2 2 MIC | 44832970 | 29828332 | 21300497 | 1958608 |
| Trimethoprim rep2 0.5 MIC | 15728504 | 10280783 | 7822460 | 1412759 |
| Trimethoprim rep2 1 MIC | 46026530 | 28353523 | 20006397 | 1834585 |
| Colistin rep1 0.25 MIC | 27876899 | 18963569 | 13428581 | 2002696 |
| Colistin rep1 2 MIC | 19433367 | 13148387 | 9812801 | 129362 |
| Colistin rep1 0.5 MIC | 28939394 | 20566623 | 15059491 | 2043438 |
| Colistin rep1 1 MIC | 24423794 | 17268018 | 12877047 | 1969568 |
| Colistin rep2 0.25 MIC | 30155446 | 20992697 | 15973349 | 2103056 |
| Colistin rep2 2 MIC | 21360661 | 15053161 | 11947299 | 213304 |
| Colistin rep2 0.5 MIC | 34797428 | 23536459 | 17730494 | 2162536 |
| Colistin rep2 1 MIC | 26900588 | 19276674 | 14773255 | 2071129 |
